# Iron Overload in Patients With Heavily Transfused Sickle Cell Disease—Correlation of Serum Ferritin With Cardiac T2^*^ MRI (CMRTools), Liver T2^*^ MRI, and R2-MRI (Ferriscan®)

**DOI:** 10.3389/fmed.2021.731102

**Published:** 2021-10-25

**Authors:** Salam Alkindi, Vinodh Panjwani, Sarah Al-Rahbi, Khalid Al-Saidi, Anil V. Pathare

**Affiliations:** ^1^Department of Hematology, Sultan Qaboos University Hospital, Muscat, Oman; ^2^College of Medicine and Health Sciences, Sultan Qaboos University, Muscat, Oman

**Keywords:** sickle cell disease, blood transfusion, iron overload, serum ferritin, cardiac T2^*^, FerriScan®, MRI

## Abstract

The treatment of sickle cell disease (SCD) is mainly supportive, except for a minority, who receive bone marrow transplantation (BMT). Serum ferritin (SF) is routinely available but is notoriously unreliable as a tool for iron-overload assessment since it is an acute-phase reactant. Although blood transfusion is one of the most effective ways to deal with specific acute and chronic complications of SCD, this strategy is often associated with alloimmunization, iron overload, and hemolytic reactions. This study, thus, aims to evaluate iron overload in patients with SCD on chronic blood transfusions and specifically, correlate SF with the current standard of care of iron-overload assessment using MRI-based imaging techniques. Amongst a historic cohort of 58 chronically transfused patients with SCD, we were able to evaluate 44 patients who are currently alive and had multiple follow-up testing. Their mean age (±SD) was 35 (9) years and comprised of 68.2% of women. The studied iron-overload parameters included cardiac T2^*^ MRI, liver iron concentration (LIC) by Liver T2^*^ MRI, and serial SF levels. Additionally, in a smaller cohort, we also studied LIC by FerriScan^©^ R2-MRI. Chronic blood transfusions were necessary for severe vaso-occlusive crisis (VOC) (38.6%), severe symptomatic anemia (38.6%), past history of stroke (15.9%), and recurrent acute chest syndrome (6.9%). About 14 (24%) patients among the original cohort died following SCD-related complications. Among the patients currently receiving chelation, 26 (96%) are on Deferasirox (DFX) [Jadenu® (24) or Exjade® (2)], with good compliance and tolerance. However, one patient is still receiving IV deferoxamine (DFO), in view of the significantly high systemic iron burden. In this evaluable cohort of 44 patients, the mean SF (±SD) reduced marginally from 4,311 to 4,230 ng/ml, mean Liver T2^*^ MRI dropped from 12 to 10.3 mg/gm dry weight, while the mean cardiac T2^*^MRI improved from 36.8 to 39.5 ms. There was a mild to moderate correlation between the baseline and final values of SF ng/ml, *r* = 0.33, *p* = 0.01; Cardiac T2^*^ MRI ms, *r* = 0.3, *p* = 0.02 and Liver T2^*^ MRI mg/kg dry weight, *r* = 0.6, *p* < 0.001. Overall, there was a positive correlation between SF and Liver T2^*^ MRI (Pearson's *r* = 0.78, *p* < 0.001). Cardiac T2^*^MRI increased with the decreasing SF concentration, showing a negative correlation which was statistically significant (Pearson's *r* = −0.6, *p* < 0.001). Furthermore, there was an excellent correlation between SF ng/ml and LIC by FerriScan^©^ R2-MRI mg/g or mmol/kg (Spearmen's rho = −0.723, *p* < 0.008) in a small subset of patients (*n* = 14) who underwent the procedure. In conclusion, our study demonstrated a good correlation between serial SF and LIC by either Liver MRI T2^*^ or by FerriScan^©^ R2-MRI, even though SF is an acute-phase reactant. It also confirms the cardiac sparing effect in patients with SCD, even with the significant transfusion-related iron burden. About 14 (24%) patients of the original cohort died over the past 15 years, indicative of a negative impact of iron overload on disease morbidity and mortality.

## Introduction

Sickle cell disease (SCD) refers to a group of inherited conditions, characterized by sickle-shaped red blood cells that precipitate recurrent episodes of vaso-occlusive episodes (VOC). The WHO published a global prevalence map of SCD and other data (http://www.who.int/genomics/public/Maphaemoglobin.pdf), revealing that about 20–25 million individuals of health organizations, worldwide, have homozygous SCD; about 12–15 million in sub-Saharan Africa, 5–10 million in India and about 3 million distributed in other parts of the world. It is estimated that 312,000 people with sickle hemoglobin (Hb) are born each year throughout the world, with the majority of these births (236,000) in sub-Saharan Africa (1), whilst, in Oman, the sickle cell gene has an overall prevalence of 6% (2). A yearly increase as the result of newborn cases is about 120–150 (3). Thus, SCD is a major public health problem in the Sultanate of Oman, with a high rate of morbidity and mortality (4–12).

Blood vessel occlusion is a fundamental pathological process in SCD (13, 14). During VOC, the vessel lumen is blocked by cells interrupting the capillary blood flow to various organs and other parts of the body. This precipitates an inflammatory process that leads to painful crises and damage to the brain, the liver, the kidneys, the lungs, the spleen, and other vital organs (15, 16). Painful crises affect virtually all patients with SCD, often beginning in late infancy and recurring throughout life (17, 18).

High-risk patients with SCD, particularly children, in accordance with the local and international guidelines, are treated with periodic on demand or chronic blood/exchange transfusions (19, 20). This reduces recurrent (21, 22) and initial stroke by over 80% (23, 24). Unfortunately, it is also associated with a high rate of complications; including the transmission of infective agents, iron overload, alloimmunization, and transfusion reactions (9, 25–27).

Alloimmunization of SCD remains a serious consequence of blood transfusions that often leads to life-threatening, acute and delayed transfusion reactions (28–30). Although allo-immunization in patients with SCD arises mostly due to RBC antigenic differences, only a subset of these patients develops RBC allo- or auto-antibodies, in spite of a similar transfusional background, indicating an underlying inherent genetic susceptibility. Genome-wide sequencing studies have shown single nucleotide polymorphisms (SNPs) on Chromosomes 2 and 5 approaching statistical significance with SNPs in CD81 gene, that encodes signal modulation of B lymphocytes, showing a strong association with alloimmunization and, thus, could serve as predictive biomarkers for alloimmunization (31).

Current guidelines recommend blood transfusions for primary and secondary prophylaxis (i.e., stroke) and therapy (i.e., acute chest syndrome and stroke) (19, 20), with less conclusive data, for other complications, such as priapism, VOCs, leg ulcers, pulmonary hypertension, and during complicated pregnancies (25, 32). Classically, serum ferritin (SF) and liver biopsy have been used to monitor patients with iron overload and assess their response to chelation therapy. SF has the advantage of being widely available, but is an acute-phase reactant, and does not always correlate with body iron stores (33). MRI has now emerged as the standard of care for effective detection and quantification of iron in the heart and the liver. T2 and T2^*^ are two approaches utilized to assess hepatic iron, based on T2 spine-echo sequences and gradient-echo sequences, respectively. Due to the increased tissue iron, the inverse of T2 and T2^*^ relaxation rates (R2 and R2^*^) are used for the quantifications of liver iron, as they are reciprocals of T2 and T2^*^, respectively and increases as iron stores increase (34). R relaxometry FerriScan® is now an FDA-approved, clinically validated, and commercially available technique for this purpose, with multiple T2 echo readings being utilized to calculate R2. R2^*^ provides a more linear correlation with liver iron concentrations (LICs). Iron deposition in patients with SCD occurs predominantly in the liver and less so in the heart and the endocrine organs (35). Hepatic deposition initially accumulates preferentially in the sinusoidal spaces; however, it generally follows the traditional pattern of transfusional iron overload, with parenchymal hepatocyte deposition also occurring early and even at low LICs (36). It is worth noting that chelation preferentially removes iron from the reticuloendotheliam (37). Nevertheless, iron-overload monitoring not only includes cardiac T2^*^MRI but also Liver T2^*^MRI as both can be obtained at the same time.

Our study aims to evaluate iron overload in patients with SCD on chronic blood transfusions and specifically correlate SF with cardiac T2^*^ MRI (CMRTools), Liver T2^*^ MRI, as well as LIC by R2-MRI (FerriScan®), with its probable impact on mortality.

## Materials and Methods

In this retrospective cohort study, transfused patients with sickle cell disease (SCD) were monitored for their iron-overload status and chelation. The study was initiated after approval from the institutional medical ethics and review committee. The indications for transfusions include acute sickle cell-related complications, or as a part of a chronic exchange transfusion program, as per local and international guidelines.

Their demographic, clinical, and laboratory data were obtained from the electronic medical records of the hospital. Baseline and current demographic information comprised of SCD diagnosis and subtype, age and gender, and indications for chronic red cell transfusion.

Serum ferritin (SF) estimations were obtained every 3 months, whereas, Liver T2^*^ MRI and Cardiac T2^*^ MRI were performed yearly, in addition to viral activity parameters (i.e., Hepatitis viruses, HIV). Almost all of these patients are currently on Deferasirox (DFX), although a few had earlier received Deferiprone (DFP) at 75 mg/kg/day in three divided doses and IV deferoxamine (DFO), especially during their intra-hospital admissions.

Assessment of iron-overload monitoring parameters included serial SF, LIC by T2^*^MRI, and Cardiac T2^*^ MRI, with data analyzed by the CMRTools software (Cardiovascular Imaging Solutions, Ltd., London) (38). The LIC was computed by Liver T2^*^ MRI (38), as well as by spin density projection-assisted R2-MRI (FerriScan^©^, Resonance Health, Australia) (39) in a smaller cohort, as per their standardized methodologies. Normal FerriScan^©^ R2-MRI reference range was 0.17–1.8 mg/g dry tissue, or 3–33 mmol/kg dry tissue (40).

### Statistical Analysis

Descriptive analyses including mean, SD, median, interquartile range (IQR), and 95% confidence intervals (CIs) were used to describe patient characteristics. Continuous variables were compared between groups using *t*-tests or Mann–Whitney U tests, as appropriate. The relationship of LIC SF was estimated by Pearson's or Spearman's correlation coefficient, as appropriate. All statistical analyses were performed with Stata12 software (Stata Corp, College Station, TX) and a *p* < 0.05 was considered statistically significant.

## Results

Our original cohort included 58 patients with a mean age (±SD) of 30 (9) years, 21 (36.2%) men, and 37 (63.8%) women. The majority, 53 (91.4%), had HbSS genotype, whereas, 5 (8.6%) had Hb Sβ^+^ Thal (Table 1). Chronic blood transfusions were indicated for severe vaso-occlusive crisis (VOC) in 24 (41.4%), severe symptomatic anemia in 19 (32.8%), history of stroke in nine (15.5%), and recurrent acute chest syndrome in six (10.3%) patients. Among these 58 patients, the baseline mean serum ferritin (SF) (±SD) was 4,092 (3,579) ng/ml, mean cardiac T2^*^MRI (±SD) was 39 ms (18), whereas mean liver T2^*^ MRI (±SD) was 12 mg/gm dry (9).

**Table 1:** Patient characteristics.

	**Baseline (*n* = 58)**	**Current (*n* = 44)**
Age, mean (±SD), yrs.	30 (9)	35 (9)
Male	21 (36.2)	14 (31.8)
Female	37 (63.8)	30 (68.2)
Genotype, *n* (%)		
SS	53 (91.4)	40 (91)
Sβ^+^ Thal	5 (8.6)	4 (9)
Transfusions, *n* (%)		
Simple	43 (74)	31 (70)
Exchange	15 (26)	13 (30)
Indication for Transfusions, *n* (%)		
Severe Crisis	24 (41.4)	17 (38.6)
Symptomatic anemia	19 (32.8)	17 (38.6)
H/o of Stroke	9 (15.5)	7 (15.9)
Recurrent ACS	6 (10.3)	3 (6.9)
Chelation, n (%)		
DFO	3 (5.1)	1 (2.3)
DFP	2 (6.9)	0 (0)
DFX	44 (75.8)	26 (59)
No chelation	9 (12.2)	17 (38.6)
Chelation dosing, mg/Kg/day		
DFO	40 mg/kg/day IV infusion	
DFP	75 mg/kg/day in divided doses	
DFX	20–40 mg/kg/day, PO	
Jadenu	12–28 mg/kg/day, PO	
Iron Assessment in the evaluable current patients (*n* = 44)		
Ferritin^a^, ng/ml, *n* (%)	44 (100)	44 (100)
Mean (±SD)	4,311 (4,030)	4,230 (3,059)
*p*-value^$^	0.9	
LIC^b^, mg/gm Dry wt, n (%)	44 (100)	44 (100)
Mean (±SD)	12 (9)	10.3 (7)
*p*-value^$^	0.1	
Cardiac T2*MRI^c^, msec, *n* (%)	44 (100)	44 (100)
Mean (±SD)	36.8 (17)	39.5 (6)
*p*-value^$^	0.1	
FerriScan R2 MRI^d^, mg/g dry tissue, *n* (%)	14 (100)	
Median (IQR)	36.5 (6.3–43)	
FerriScan R2 MRI^e^, mmol/kg dry tissue, *n* (%)	14 (100)	
Median (IQR)	658.5 (113–770)	

$ *Paired student's t-test*.

a *SF Ref. Range (10–28)*.

b LIC T2^*^ MRI Ref. Range (0.17–1.8).

c Cardiac T2^*^ MRI Ref. Range (>20 msec).

d *FerriScan^©^ R2 MRI mg/g dry tissue Ref. Range (0.17–1.8)*.

e *FerriScan^©^ R2 MRI, mmol/kg dry tissue Ref. Range (3.0–33)*.

*DFX, Deferasirox; DFP, Deferiprone; DFO, Deferoxamine (Desferal); IQR, Interquartile range; msec, milliseconds*.

In the current evaluable cohort of 44 patients with sickle cell disease (SCD) (Table 1), the mean SF (±SD) reduced marginally from a baseline value of 4,311 (4,030) ng/ml to 4,230 (3,059) ng/ml. Mean LIC (±SD) by Liver T2^*^ MRI dropped marginally from 12 (9) to 10.3 (7) mg/gm dry wt. Mean Cardiac T2^*^ MRI (±SD) improved marginally from 36.8 (17) to 39.5 (6) ms. The mean time (±SD yrs) between the first and the last examinations was 3 (±2) years.

**Figure 1:**
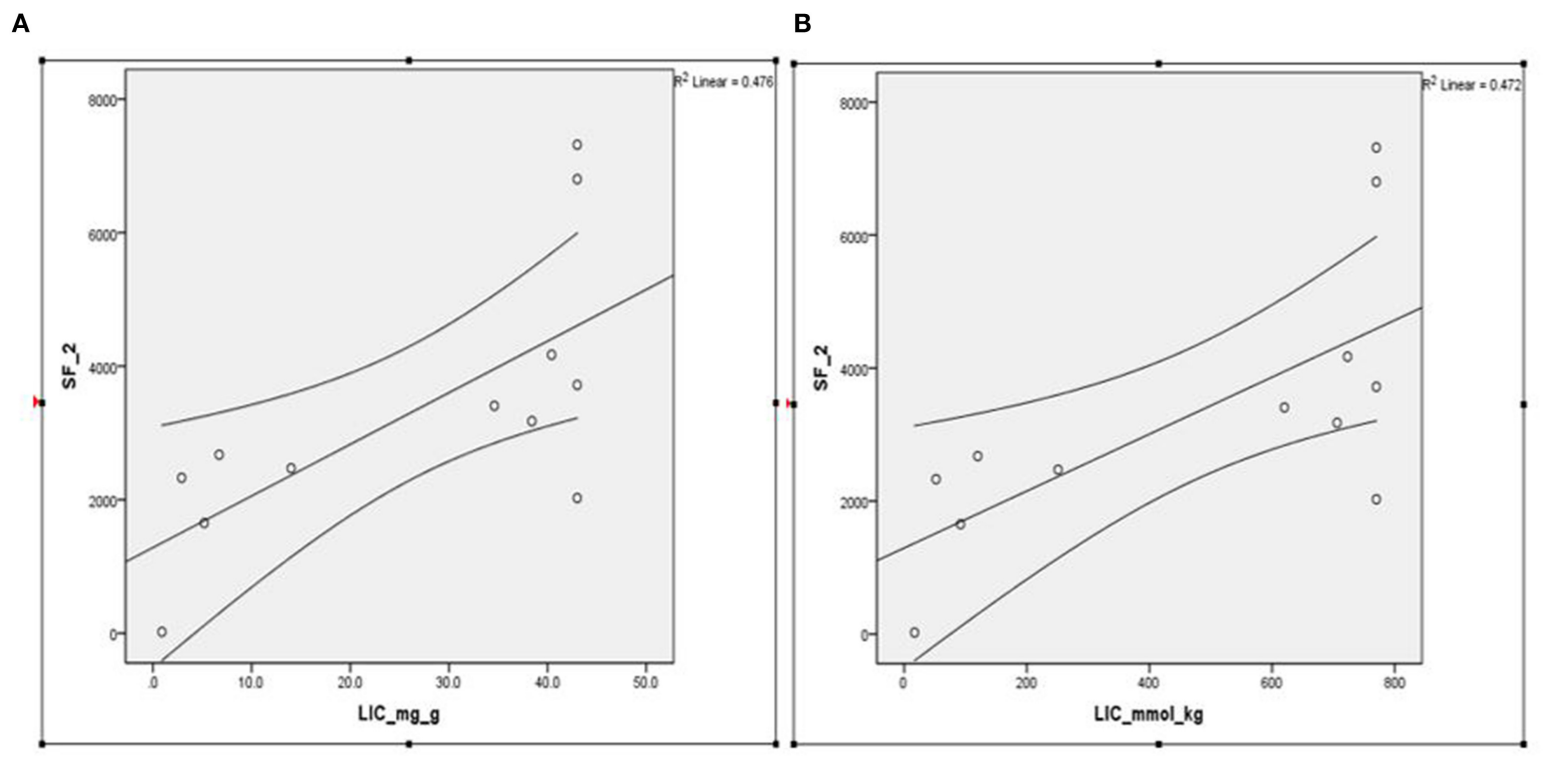
**(A,B)** Spearmen's correlation between SF and LIC R2 by FerriScan^©^ as either **(A)** mg/kg or **(B)** mmol/kg dry tissue showing that LIC by R2 MRI (FerriScan^©^) improves with decreasing SF concentrations (Spearmen's rho = −0.723, *p* < 0.008). SF, serum ferritin; LIC, liver iron concentration.

Serial SF data analyzed at the time of performing FerriScan® iron study on a subset of these patients (*n* = 14) revealed a median SF of 2,926 ng/ml, with an interquartile range (IQR) between 2,119 and 3,676 and showed an excellent correlation with LIC mmol/kg as well as mg/kg dry tissue (Spearmen's rho = 0.723, *p* < 0.008; Figures 1A,B). Further, the median LIC by FerriScan^©^ R2-MRI analysis was 36.5 mg/gm dry tissue weight, with an IQR between 6.3 and 43. Alternatively, in terms of mmol/kg of dry tissue weight, the median LIC was 658.5 with an IQR between 113 and 770.

44 patients are alive and had multiple follow-up investigations, whereas 14 (24%) among the original cohort, have died owing to SCD-related complications. The mean age (±SD) of this cohort is 35 (9) years, with a range between 20 and 64 years, comprising of 30 (68.2%) women (Table 1). Currently, 27 (61.4%) patients are receiving iron chelation therapy. Among those on chelation, 26 (96%) are receiving Deferasirox (DFX) [Jadenu® (24), or Exjade® (2)], with good compliance and tolerance. However, one patient still needs parenteral Deferoxamine (DFP) (Desferal®) in view of the very high systemic iron burden. The remaining 17 patients currently are not on any chelation therapy but are followed and monitored regularly, to check the need for the introduction of chelation. It is our policy to stop chelation once the SF levels reach below 500 ng/ml on two consecutive quarterly follow-ups.

Table 2 shows the effect of chelation when analyzing the progressive change in iron burden characterized, as overall, on demand and regular transfusion subgroups. There were no statistically significant differences in the serial SF (ng/ml), Liver T2^*^ MRI (mg/kg dry wt.), and cardiac T2^*^ MRI (ms), in the subgroups receiving on demand blood transfusions (*n* = 31) or regular transfusions (*n* = 13).

**Table 2:** Effect of chelation: progressive change in iron burden with overall, on demand, and regular transfusion subgroups.

	**Initial serial SF, ng/ml mean (±SD)**	**Final serial SF, ng/ml mean (±SD)**	Initial LIC T2^*^ MRI, mg/kg dry wt. mean (±SD)	Final LIC T2^*^ MRI, mg/kg dry wt. mean (±SD)	Initial Cardiac T2^*^ MRI, ms mean (±SD)	Final Cardiac T2^*^ MRI, ms mean (±SD)
Overall (*n* = 44)	4,311 (4,030)	4,230 (3,059)	12 (9)	10.3 (7)	36.8 (17)	39.5 (6)
*p*-value^$^	0.9		0.1		0.1	
On Demand (*n* = 31)	4,436 (4,273)	4,389 (2,168)	11 (8)	9.5 (4)	37.5 (17)	39.4 (4)
*p*-value^$^	0.9		0.4		0.6	
Regular (*n* = 13)	4,030 (2,429)	3,875 (3,183)	16 (9)	12.5 (8)	37 (26–47)	40.1 (7)
*p*-value^$^	0.9		0.2		0.1	

$ *Paired Student's t-test*.

Table 3 shows the correlation of LIC with serial SF in specific subgroups characterized by the initial baseline and final current values when correlated with the three different levels of LICs. There was no statistically relevant trend in the Pearson's correlations between Liver T2^*^ MRI (mg/kg dry wt.) and SF (mg/ml) subgroups characterized by LIC < 7 with SF < 1,500, LIC between 7 and 15 with SF between 1,500 and 2,500, and LIC > 15 with SF > 2,500 among the initial and final values. However, the only subset that showed a good correlation was with the lowest SF and LIC levels, where Pearson's correlation improved from *r* = 0.2 to *r* = 0.86 (*p* = 0.002).

**Table 3:** Effect of chelation: correlation of liver iron concentration (LIC) with serial serum ferritins in specific subgroups, initial baseline values, and final current values.

**Initial baseline**	**Median (95%CI)**	**Pearson's correlation, *r***	***p*** **-value**
Serial Serum Ferritin subgroups, <1,500 ng/ml	1,206 (540–1,464)	0.2	0.32
LIC by T2^*^ MRI subgrouping, <7 mg/kg dry wt.	4 (2–6.9)		
Serial Serum Ferritin subgroups, 1,500–2,500 ng/ml	1,990 (1,776–2,077)	0.4	0.08
LIC by T2^*^ MRI subgrouping, 7–15 mg/kg dry wt.	11 (8–13)		
Serial Serum Ferritin subgroups, >2,500 ng/ml	4,573 (2,878–22,244)	0.5	0.06
LIC by T2^*^ MRI subgrouping, >15 mg/kg dry wt.	22 (16–39)		
Current/Final			
Serial Serum Ferritin subgroups, <1,500 ng/ml	553 (27–1,445)	0.86	0.002
LIC by T2^*^ MRI subgrouping, <7 mg/kg dry wt.	2.1 (1.5–3.8)		
Serial Serum Ferritin subgroups, 1,500–2,500 ng/ml	1,674 (1,637–2,256)	0.1	0.6
LIC by T2^*^ MRI subgrouping, <7–15 mg/kg dry wt.	10 (7.8–14.4)		
Serial Serum Ferritin subgroups, >2,500 ng/ml	5,365 (2,691–15,083)	0.3	0.2
LIC by T2^*^ MRI subgrouping, >15 mg/kg dry wt.	19 (15.5–28)		

*CI, Confidence Interval*.

**Figure 2:**
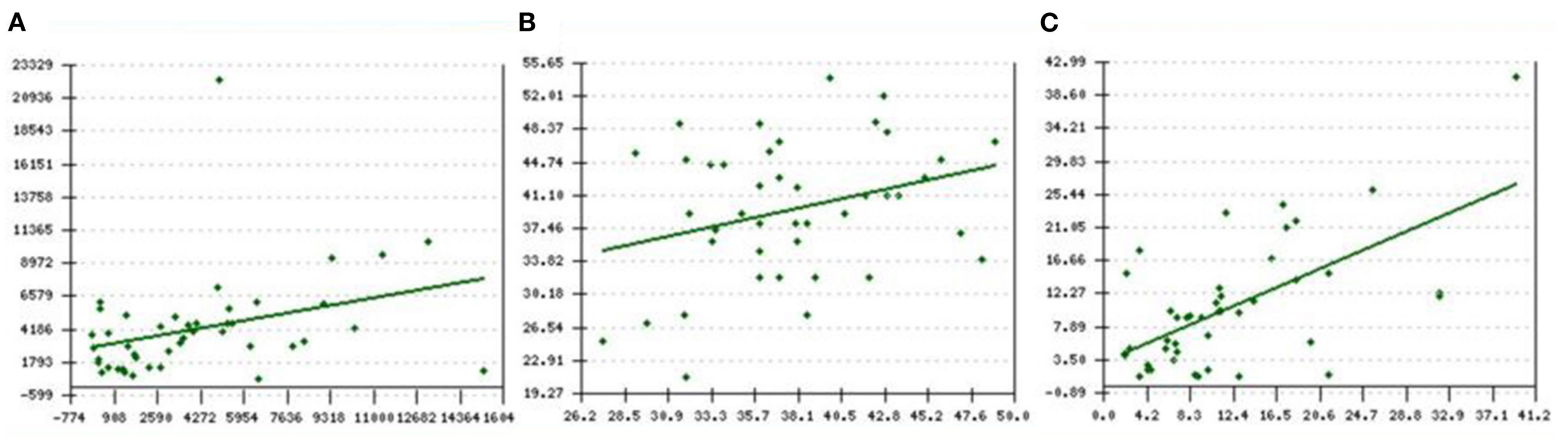
**(A–C)** Pearson's correlation between baseline and final values of **(A)** SF ng/ml, *r* = 0.33, *p* = 0.01 **(B)** Cardiac T2* MRI ms, *r* = 0.3, *p* = 0.02 and **(C)** LIC T2* MRI mg/kg dry weight, *r* = 0.6, *p* < 0.001. X-axis represents the baseline levels of respective parameters vs. y-axis which represents the final/current levels of the same parameter. SF, serum ferritin; LIC: liver iron concentration.

Figures 2A–C shows a mild to moderate Pearson's correlation between the baseline and final values of (A) SF (ng/ml), *r* = 0.33, *p* = 0.01 (B) Cardiac T2^*^ MRI (ms), *r* = 0.3, *p* = 0.02 and (C) Liver T2^*^ MRI (mg/kg dry weight), *r* = 0.6, *p* < 0.001.

**Figure 3:**
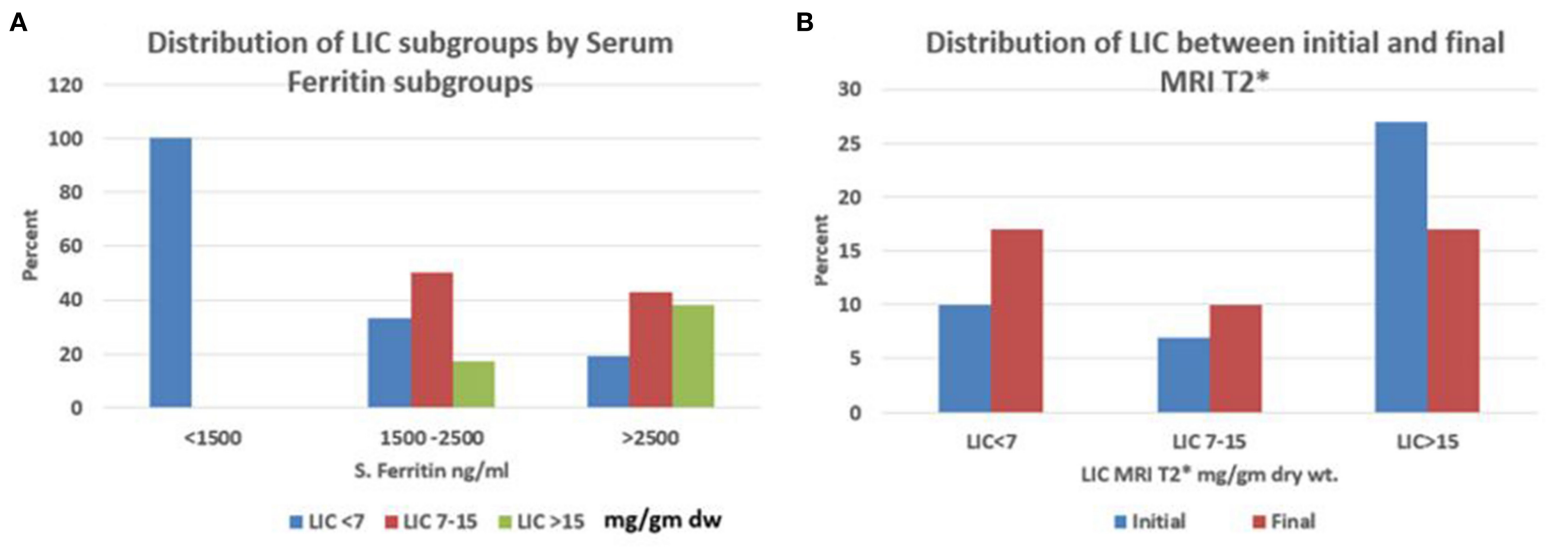
**(A,B)** Percentage distribution of **(A)** LIC subgroups ≤7, 7–15, ≥15, mg/kg dry tissue by SF subgroups ≥1,500, 1,500–2,500 and ≥2,500 ng/ml and; **(B)** LIC subgroups ≤7, 7–15, ≥15, mg/kg dry tissue between the initial and final MRI T2^*^. SF, serum ferritin; LIC: liver iron concentration.

Figure 3A shows the percentage distribution of serum SF (ng/ml) subgroups <1,500, between 1,500 and 2,500, and >2,500, with respect to the three Liver T2^*^ MRI subgroups (mg/kg dry wt.) <7, between 7 and 15, and >5, respectively.

Figure 3B shows the percentage distribution of Liver T2^*^ MRI (mg/kg dry wt.) subgroups <7, between 7 and 15, and >15 among the initial and final values.

**Figure 4:**
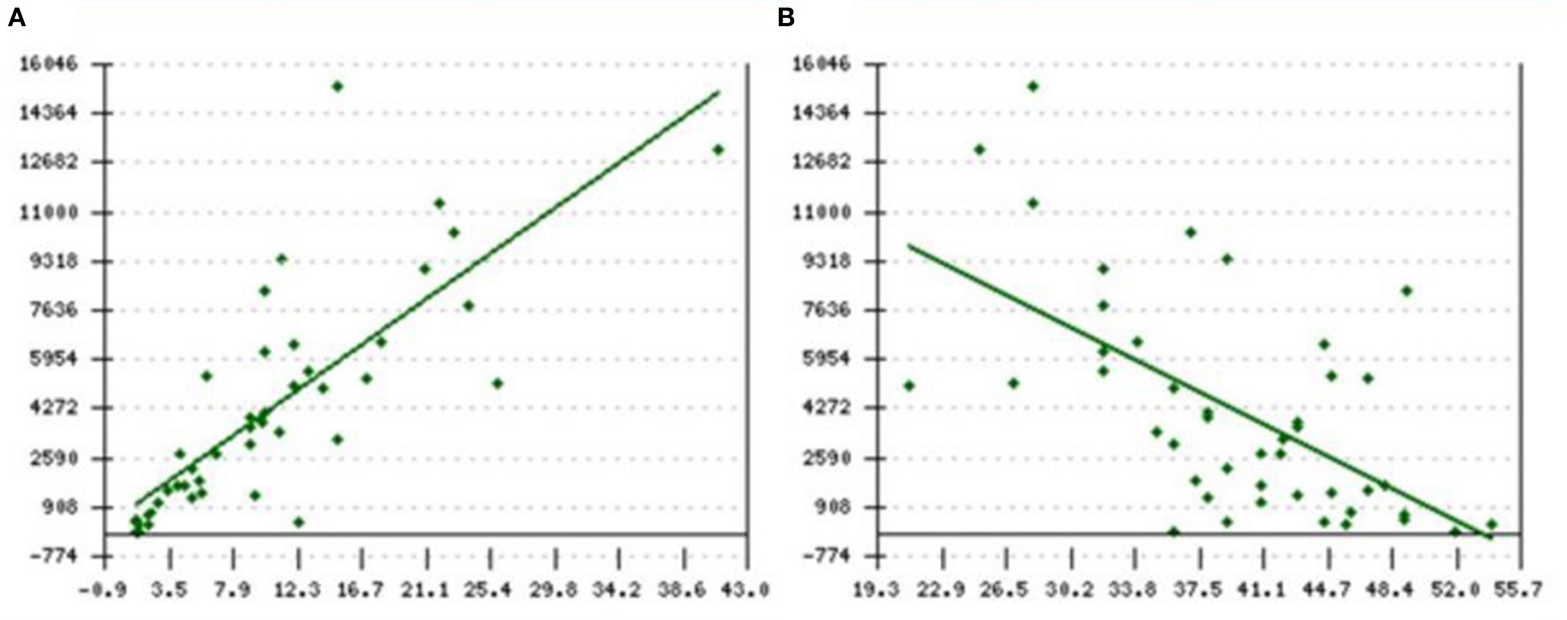
**(A,B)** Pearson's correlation between current **(A)** SF and Liver T2*MRI (positive) *r* = 0.78; *p* < 0.001 as well as **(B)** SF and Cardiac T2*MRI (negative) *r* = −0.6; *p* < 0.001. X-axis represents final/current LIC levels whereas SF and T2* are represented on Y-axis in **(A,B)**, respectively. SF, serum ferritin; LIC, liver iron concentration.

Figure 4A shows a positive correlation between the serial SF and Liver T2^*^ MRI showing that Liver T2^*^MRI reduced with decreasing SF concentration, and this change was statistically significant (Pearson's *r* = 0.78, *p* < 0.001).

Figure 4B shows that Cardiac T2^*^MRI increased with the decreasing SF concentration, showing a negative correlation, which was also statistically significant (Pearson's *r* = −0.6, *p* < 0.001). Importantly, none of these patients had any evidence of cardiac iron overload with the mean cardiac T2^*^ MRI levels of 36.8 ms at the initial examination which improved to 39.5 ms in the final examination. Further, the range of cardiac T2^*^ MRI varied from 21 to 54 ms, which was always above the normal reference range of >20 ms (CMRTools software).

Supplementary Figures 1, 2 illustrate the sample images of FerriScan^©^ R2-MRI and Cardiac T2^*^ MRI of a putative patient showing liver iron overload, but sparing the heart.

Supplementary Figures 3, 4 show sample images of Liver T2^*^ MRI and Cardiac MRI T2^*^ of a putative patient showing liver iron overload, but sparing the heart.

## Discussion

In 2006, the WHO recognized hemoglobinopathies, including sickle cell disease (SCD), as a global public health problem and Oman has a high prevalence of hemoglobin (Hb) disorders (3) (http://apps.who.int/iris/bitstream/handle/10665/20890/A59_9-en.pdf?sequence=1&isAllowed=y).

In SCD, blood transfusions remain a critical therapeutic intervention as it improves the blood flow by reducing the proportion of red blood cells capable of sickling (25). Further, the chronic hemolytic state that is characteristic of SCD, with the release of free heme that quenches nitrous oxide and the activation of inflammatory cascades, ultimately results in hemolysis and endothelial damage (14, 41). Blood transfusions will not only limit the amount of hemolysis but also prevent the endothelial damage that results in the high proportions of sickle polymer-containing red blood cells. Additionally, blood transfusions will also increase the blood oxygen-carrying capacity in severe chronic anemia or with severe vaso-occlusive episodes (VOC). Therefore, blood transfusions are established not only as a preventive strategy for stroke, especially in patients with high intracranial blood flow velocity revealed by transcranial Doppler studies, but also for their therapeutic benefits in a wide variety of complications, such as VOC, priapism, pulmonary hypertension, and during complicated pregnancies (25). However, the major and unavoidable complication of blood transfusions in SCD is systemic iron overload. In our setup, on demand blood transfusions accounted for almost two-thirds (70–74%) of these patients, whereas, regular transfusions were given in less than a third of these patients (26–30%). This proportion is significant since it represents the current real-life situation in patients with SCD who receive chronic blood transfusions.

Repeated transfusions of packed red blood cells (PRBCs) are currently the simplest and the primary method employed in chronic transfusion programs (42). However, although apheresis of RBC (erythrapheresis) is currently the safest and the most efficient method, it is costly, complicated, and cannot be implemented everywhere, nor is it suitable for all patients (42). Therapeutic phlebotomy is an alternate technique that is safe and well-tolerated, with net iron removal but is currently used only in patients with SCD who have iron overload and have undergone bone marrow transplantation (BMT) for SCD (43). We have used this method in eight of the 58 patients from the original cohort who underwent BMT. It has given us good results in terms of managing iron overload in this setting, but three patients in this cohort expired due to post-BMT complications, while the remaining five patients are doing well with regular monitoring of their iron-overload status. Manual exchange transfusions combined with one or more manual phlebotomies with a PRBC transfusion is, thus, what we have been practicing in the majority of our patients on chronic blood transfusions. However, iron overload is the principal side effect of this therapy.

The utility of the SF alone, while monitoring iron overload in the chronically transfused patients with SCD is disputable. This is so because of the propensity of the inflammatory stimulus that would invariably, falsely elevate SF. However, using serial SF estimations, performed during steady-state, will help in the assessment of the true systemic iron-load status as was seen in our current practice. Routinely performing SF estimations every quarter, enabled us to ascertain the average SF levels over the year, and avoided the spikes seen during acute inflammatory states like VOCs. It, thus, became a reliable tool in the monitoring of iron-overload status in this cohort as it showed a fairly good correlation with Liver T2^*^ MRI results (Figure 4A). There was a progressive fall in the percentage of LIC > 15 with a rise in the LIC < 7 indicative of the progressive improvement in the iron-overload status over the follow-up years (Figure 3B). Further, in this cohort, SF levels below 1,500 ng/ml and LIC levels below <7 mg/kg dry wt. showed a good correlation, but SF levels above 1,500 and >2,500 ng/ml were associated with wide variability in the Liver T2^*^ MRI (Table 3, Figure 3A). These findings are not only consistent with other prior studies (44–46), but also point to the progress in patient management that historically did not receive optimal monitoring iron overload (47). Thus, with the long-term aim of getting the Liver T2^*^ MRI below 7 mg/kg, the utility of annual LIC measurement with T2^*^ MRI or FerriScan^©^ R2-MRI, where available, needs to be emphasized, especially when the SF levels are above 2,500 ng/ml. A significant reduction in Liver T2^*^ MRI between the first and final liver MRI studies with a greater proportion of LIC below 7 mg/g dry wt. supports the putative benefit from Liver T2^*^ MRI monitoring (Figure 3B). Further, a clear positive correlation between SF and Liver T2^*^ MRI consolidates this point (Figure 4A, Pearson's *r* = 0.78; *p* < 0.001). However, ferritin is a poor indicator and although ferritin is a convenient measure of iron status, ferritin trends are unable to predict changes in LIC in individual patients (48). Ferritin trends need to be interpreted in conjunction with the direct measurement of LIC.

Tissue iron is paramagnetic and increases the MRI relaxation rates, R2 and T2^*^ in a quantifiable manner (32, 39). These non-invasive iron estimation techniques by MRI have been validated at several centers (32, 38, 39, 46). Our liver FerriScan^©^ R2-MRI study on a small subset of patients gave us a comparative view of the two currently available liver tissue iron estimation techniques. Further, since the FerriScan^©^ R2-MRI platform was not available locally, over the years, we have been relying consistently on the 1.5 Tesla MRI T2^*^ technique, after purchasing a license for the use of the CMRTools software. This certainly has positively affected the clinical care in our patient population, with Liver T2^*^ MRI helping us to make the necessary adjustments in chelation therapy. The results also served to reinforce the continuation of the current assessment tools to optimize patient care and monitor the iron overload to address adherence to chelation therapy and improve management decisions with increasing familiarity with this technology. Moreover, we have discontinued iron chelation therapy in 12 patients, as their SF levels were consistently lower than 500 ng/ml, and they are meticulously monitored by serial SF levels and Liver T2^*^MRI results. The other five post-BMT patients who are not on chelation therapy are on periodic phlebotomies with a progressive decline in the SF and Liver T2^*^ MRI levels with regular follow-up monitoring.

Patients with transfusion-dependent anemia develop fatal cardiac and endocrine toxicities from iron overload (32, 49). Further, although blood transfusion therapy is life-saving for patients with SCD and thalassemia, iron overload (especially cardiac) has impacted significantly on survival, especially before the era and availability of chelation therapy (50). However, there are intrinsic differences between patients with SCD and thalassaemia in terms of the sites of parenchymal organ involvement. This is because it is believed that patients with thalassemia have increased plasma malondialdehyde and circulating non-transferrin bound iron (NTBI) relative to patients with SCD, and lower levels of some cytokines (interleukin 5 and interleukin 10) and γ-tocopherol (36). Thus, in contrast to hemolytic anemias like Thalassaemia and SCD, in patients with Diamond Blackfan syndrome, there is variable ineffective erythropoiesis, with little or no RBC production. Thus, iron-related toxicity in this situation results from unutilized iron from senescent red cells that are not reused and much of it becomes labile plasma iron and then labile cellular iron, causing toxic damage (51).

These significant differences also support the hypothesis that the biology of SCD predominantly shows an increased inflammation, with elevated interleukin-6 and increased levels of protective antioxidants compared to patients with thalassemia, in whom NTBI-related parenchymal damage results in organ failure (36, 37). Thus, although iron deposition in patients with SCD generally follows the traditional pattern of transfusional iron overload, with parenchymal hepatocyte iron overload, optimal chelation therapy is desirable to offset the continuous iron deposition in the liver parenchyma. Our study confirmed the role of iron chelation and monitoring showing the cardiac sparing effect in patients with SCD, even with significant transfusion of burden-related systemic tissue iron overload (Table 1 and Supplementary Figures 1–4). This is further substantiated with a clear negative correlation between SF and cardiac T2^*^MRI (Pearson's *r* = −0.6; *p* < 0.001, Figure 4B). However, to further optimize iron chelation, progressive reduction in SF and LIC are the desirable goals of therapy to bring down the total body iron burden. We need to pursue this objective in order to reduce the risk of long-term complications of liver iron overload, namely liver cirrhosis and carcinoma, which are known to occur in the long term. Thus, as in the management of Thalassaemia Major, our aim is to try to further optimize chelation and to achieve not only a clear heart but also try to get and maintain the LIC below 3 mg/kg dry wt.

Although deferoxamine (DFO) has been historically the major iron-chelating therapy of transfusional iron overload, compliance is a major hindrance in achieving the optimal therapeutic goal. Further, the availability of oral iron chelation with Deferiprone (DFP) since 1987 was useful, but showed poor efficacy, when used alone as compared to DFO. However, Deferasirox (DFX) became clinically available in 2006 and has been the preferred method that was adopted in our hospital for chelation therapy. DFX is the predominately prescribed chelating agent in our cohort, with occasional use of DFO during hospital admissions. Due to the small numbers of subjects receiving DFP and DFO, a direct comparison of the change in the iron burden on different chelating agents is not possible. Moreover, currently, no patient is on DFP therapy. Nevertheless, good adherence with the oral chelator agent correlated with better SF and Liver T2^*^ MRI results. This supports the need for routine assessment of adherence and barricades to it. The success of chelation therapy is significantly impacted by patient adherence to the prescribed treatment, and consequently, adjustment of drug schedules for increasing the adherence to treatment becomes critical (52). Our patients were fortunate to have been able to receive oral iron chelation with DFX, as although this is a relatively expensive treatment, and the patients do need to continue the same over long periods or indefinitely, they also continue to receive chronic blood transfusions. This oral iron chelation therapy is completely free of cost to Omani patients since the treatment cost is borne by the government healthcare providers. Thus, almost all the current patients on oral iron chelator are receiving DFX therapy with good tolerance and compliance. It has been owing to the good compliance that we were able to take 12 patients off chelation therapy and they are currently being monitored with periodic SF levels and T2^*^ MRI imaging results.

Limitations of this study include the retrospective data analysis in this study cohort, who received chronic blood transfusions for various indications. LIC by FerriScan^©^ R2-MRI is not widely available, which limits its use as an assessment platform, and since we did not have it locally, we had to develop Liver and Cardiac T2^*^ MRI technique and get the license for the use of the CMR tools software to compute LIC T2^*^ MRI and Cardiac T2^*^ MRI. The effectiveness of erythrocytapheresis in preventing or reducing systemic iron loading in patients with SCD could not be assessed as it was not routinely available at our center, but it is a technique that can be effectively used in patients who are on chronic blood transfusion therapy. Phlebotomy has been useful in the eight patients with SCD who were transplanted, but its use was restricted only to this category of patients. Finally, mortality was seen in 14 patients (24%) being multifactorial, could not be assessed to see a direct cause and effect relationship to the iron overload. Three patients died due to post-BMT complications while two died with chronic renal failure. The remaining nine patients died due to multiorgan failure following sepsis. Certainly, there was a significant background for iron overload in these patients at the time of death which could have precipitated the complications that ultimately culminated in the death of these patients.

In summary, the availability of oral chelation paralleled with the assessment of iron overload by MRI imaging has improved the management of iron overload in our population with SCD. Further, although SF has significant limitations in the assessment of iron burden when performed repeatedly and in steady-state, it showed a good statistically significant correlation with LIC and cardiac iron in patients with SCD. Moreover, it further needs to be emphasized that these assessment tools are essential for the optimal management of iron overload. Additionally, judicious limitation of unnecessary simple transfusion therapy, wherever possible, and encouraging adherence to chelation therapy are also important strategies to control iron overload and its associated clinical consequences.

## Data Availability Statement

The original contributions presented in the study are included in the article/Supplementary Material, further inquiries can be directed to the corresponding author.

## Ethics Statement

Ethical review and approval was not required for the study on human participants in accordance with the local legislation and institutional requirements. Written informed consent for participation was not required for this study in accordance with the national legislation and the institutional requirements.

## Author Contributions

SA, VP, SA-R, and KA-S were fully involved in the conception and design of the study, recruitment and care of patients, and acquisition of data. AP and SA have been fully involved in the analysis and interpretation of data and were instrumental in the drafting the article and critical appraisal before submission. All authors have made substantial contributions and have seen and approved the final version of the manuscript.

## Funding

The FerriScan study on a small subset of patients was funded by a generous grant from Novartis Ltd.

## Conflict of Interest

The authors declare that the research was conducted in the absence of any commercial or financial relationships that could be construed as a potential conflict of interest.

## Publisher's Note

All claims expressed in this article are solely those of the authors and do not necessarily represent those of their affiliated organizations, or those of the publisher, the editors and the reviewers. Any product that may be evaluated in this article, or claim that may be made by its manufacturer, is not guaranteed or endorsed by the publisher.

## Supplementary Material

Supplementary Figures 1–4 Sample Images (1 & 2) of LIC by FerriScan^©^ and LIC and Cardiac T2^*^ MRI of a patient showing liver iron overload but sparing the heart. Sample Images (3 & 4) of LIC by MRI T2^*^ and Cardiac MRI T2^*^ of a patient showing liver iron overload but sparing the heart. SF, serum ferritin; LIC, liver iron concentration.
