# Supplementary figures and images for: Iron Overload in Patients With Heavily Transfused Sickle Cell Disease—Correlation of Serum Ferritin With Cardiac T2* MRI (CMRTools), Liver T2* MRI, and R2-MRI (Ferriscan®)
Source: Front Med (Lausanne). 2021 Oct 25;8:731102. doi: 10.3389/fmed.2021.731102 (PMC8573209; doi:10.3389/fmed.2021.731102)

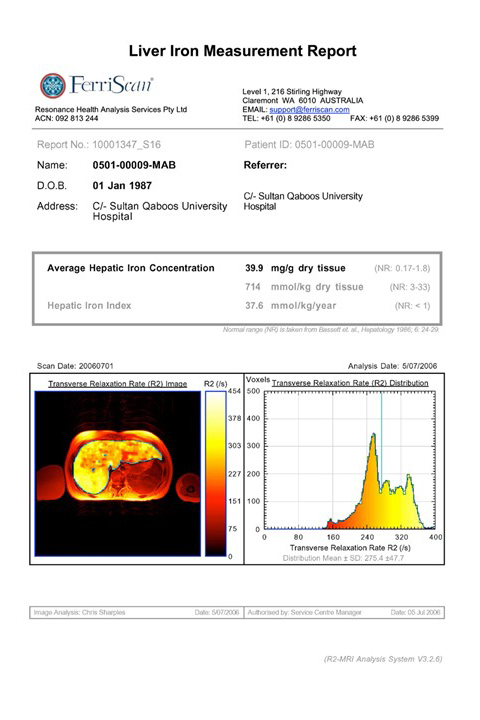

Supplement: Supplementary file 1 [file Image_1.JPEG]

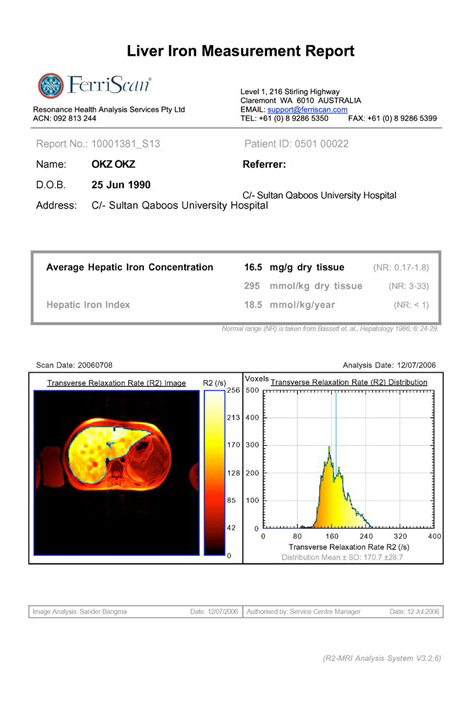

Supplement: Supplementary file 2 [file Image_2.JPEG]

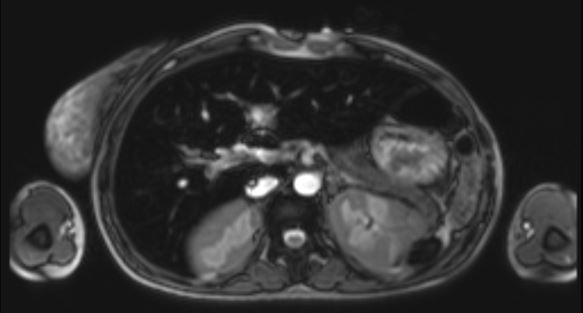

Supplement: Supplementary file 3 [file Image_3.JPEG]

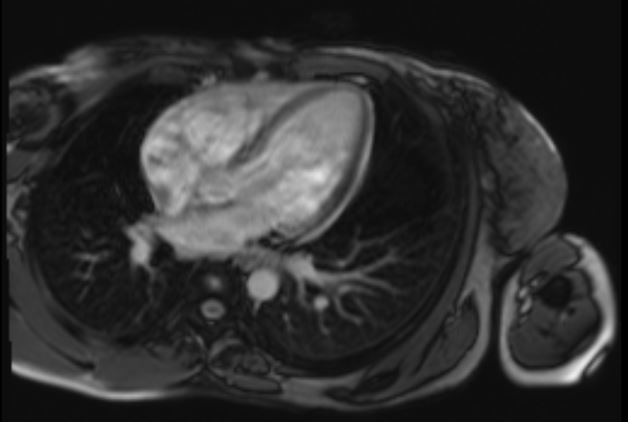

Supplement: Supplementary file 4 [file Image_4.JPEG]

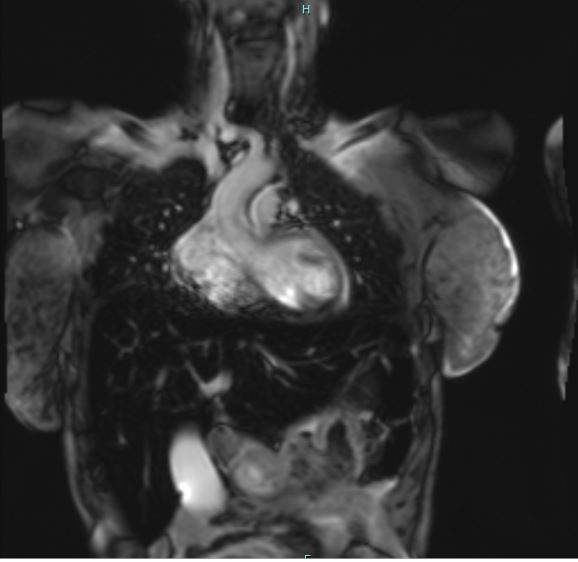

Supplement: Supplementary file 5 [file Image_5.JPEG]

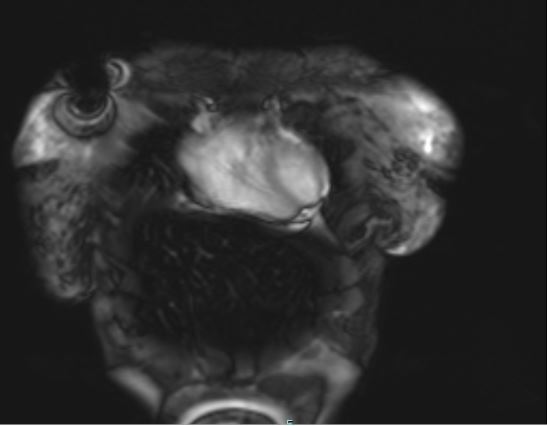

Supplement: Supplementary Figures 1–4 — Sample Images (1 & 2) of LIC by FerriScan© and LIC and Cardiac T2* MRI of a patient showing liver iron overload but sparing the heart. Sample Images (3 & 4) of LIC by MRI T2* and Cardiac MRI T2* of a patient showing liver iron overload but sparing the heart. SF, serum ferritin; LIC, liver iron concentration. [file Image_6.JPEG]
